# The prevalence of mismatch repair deficiency in ovarian cancer: A systematic review and meta‐analysis

**DOI:** 10.1002/ijc.34165

**Published:** 2022-07-06

**Authors:** Amit Atwal, Tristan Snowsill, Marcus Cabrera Dandy, Thomas Krum, Claire Newton, Dafydd Gareth Evans, Emma J. Crosbie, Neil A. J. Ryan

**Affiliations:** ^1^ Clinical Medical School University of Bristol Bristol UK; ^2^ Health Economics Group, University of Exeter Medical School University of Exeter Exeter UK; ^3^ The Lancashire Women's and Newborn Centre Burnley General Hospital East Lancashire UK; ^4^ Department of Obstetrics and Gynaecology St. Michaels Hospital Bristol UK; ^5^ Division of Evolution and Genomic Medicine, St. Mary's Hospital University of Manchester Manchester UK; ^6^ Division of Cancer Sciences, Faculty of Biology, Medicine and Health University of Manchester, St. Mary's Hospital Manchester UK; ^7^ The Academic Women's Health Unit, Translational Health Sciences, Bristol Medical School University of Bristol Bristol UK

**Keywords:** biomarkers, checkpoint inhibition, germline testing, immune therapy, immunohistochemistry, Lynch syndrome, microsatellite instability, mismatch repair, ovarian cancer, somatic testing

## Abstract

Ovarian cancer (OC) is the least survivable gynecological malignancy and presents late. Five‐year survival for OC is around 45% increasing the need for innovative treatments. Checkpoint inhibitors have shown significant clinical efficacy in mismatch repair deficient (MMRd) cancers and could be a powerful treatment in OC. However, their application in OC is limited due to the lack of data on the prevalence of MMRd. The aim of our study was to conduct a systematic review of the literature and meta‐analysis to provide an accurate estimate of the prevalence of MMRd in OC. We followed PRISMA guidelines throughout. Studies were identified by electronic searches of Medline, Embase, Cochrane CENTRAL and Web of Science followed by citation searching. Studies not written in English were excluded. All studies were reviewed by at least two independent reviewers. Proportions of test positivity were calculated by random and fixed‐effects meta‐analysis models. *I* ^2^ score was used to assess heterogeneity across studies. In total 54 studies were included with 17 532 analyzed for MMRd. The overall proportions of MMRd by immunohistochemistry and microsatellite instability analysis were 6.7% and 10.4%, respectively. MMRd was reported in all histotypes of epithelial OC but was most common in endometrioid OC. We estimate that on average 46.7% (95% CI: 28.8‐65.4) of ovarian carcinomas showing MMRd by IHC had a germline path_MMR variant identified. OC in those with Lynch syndrome seems to present at an earlier age and stage. Studies however were generally of low quality and there was a high degree of heterogeneity. A significant minority (up to 16%) of OC displays MMRd and, therefore, could be amenable to checkpoint inhibition therapy. However, the current literature base is of limited quality and therefore high‐quality prospective studies exploring MMRd in OC with the use of multimodal testing are required. In addition, trials researching efficacy of checkpoint inhibition in MMRd OC are needed.

## Graphical abstract

What's new?

Despite gains in ovarian cancer survival, overall prognosis remains poor. Thus, to further improve patient outcomes', efforts are being made to identify cancers amenable to targeted therapeutics. Ovarian cancers with a mismatch repair deficiency (MMRd) phenotype could respond to immotherapy and therefore defining the prevelance of MMRd in ovarian cancer is important. In the present meta‐analysis examining the prevalence of MMRd in ovarian cancer we found a significant minority of  ovarian cancers are MMRd. MMRd was observed in all histotypes but was more common in endometrioid tumors. Knowledge of MMRd prevalence in ovarian cancer could help guide therapeutic decisions, particularly surrounding the use of checkpoint inhibitors which are most effective in MMRd cancers.

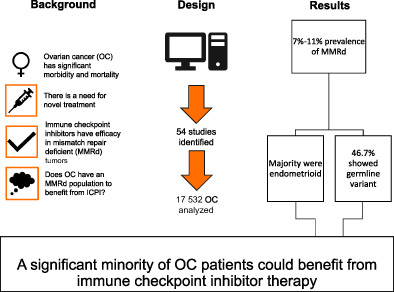

**Abbreviations**
FDA: US‐Federal Drug Administration
ICPI: immune checkpoint inhibitors
IHC: immunohistochemistry
MMR: mismatch repair
MMRd: mismatch repair deficient
MSI: microsatellite instability analysis
MSI‐H: microsatellite instability high
MSS: microsatellite stable
OC: ovarian cancer
path_MMR: pathogenic variant in a mismatch repair gene
QUADAS 2: quality and applicability of diagnostic accuracy studies
TMA: tissue microarrays

## 1 INTRODUCTION

Ovarian cancer (OC) is the least survivable gynecological malignancy in developed nations.^1^ It is associated with significant morbidity and mortality, with 230 000 women being diagnosed, and 150 000 women dying from OC annually.^1^ Survival at 5 years is less than 50% with survival rates having only increased by 30% since the mid‐1970s.^2^ The current treatment for OC consists of surgery to optimally debulk the disease alongside (neo)adjuvant platinum‐based chemotherapy with the selective addition of antiangiogenesis inhibitor bevacizumab and/or poly(ADP‐ribose) polymerase inhibitors.^1^ Numerous factors contribute to the high mortality rate associated with OC. In the first instance, the symptoms of OC are vague, and women often present with advanced disease.^2^ To date, there is no effective screening program.^3^ Finally, until recently, effective treatment innovation has been lacking.^4^

One such treatment innovation is the use of immune checkpoint inhibitors (ICPIs) in cancers with a mismatch repair deficient (MMRd) phenotype. MMRd cancers are highly immunogenic because of the production of numerous neopeptides due to their hypermutated genome.^5^ Therefore, MMRd cancers must undergo immunoediting in order to escape the immune surveillance and destruction.^6^ Immunoediting involves three steps: elimination, equilibrium and escape, with the later step in MMRd cancers often involving co‐signaling pathways; namely signaling via the PD‐1/PD‐L1 pathway.^7^ This is a druggable pathway with the use of ICPIs.

ICPIs are monoclonal antibodies that reinvigorate the antitumor immune response by targeting co‐inhibitory receptors.^8^ They bind directly to T cells inhibiting their ability to communicate with their immune checkpoint ligands. This instigates two outcomes, first, without the influence of inhibitory signals, the T cell can resume its effector functions and second, natural opsonization of antibody bound T‐cells allows for the expansion of new tumor‐specific T cells.^9^ Trial data, most notably Dung et al, demonstrated the therapeutic affinity ICPIs possesses towards MMRd cancers.^10^ Both objective response rates and progression free survival were significantly greater in MMRd cancers compared to MMR proficient cancers treated with ICPIs. These data led to the US‐Federal Drug Administration (FDA) approving Nivolumab and Pembrolizumab for use in MMRd tumors, regardless of histology and cancer site.^11^ This was the first time a cancer treatment had been approved based on a molecular characteristic.

Data regarding use of ICPIs in OC is limited. Studies have been small and given mixed results as to ICPIs efficacy in OC.^12^, ^13^, ^14^, ^15^, ^16^ However, these studies did not select for MMRd OC and therefore their inability to find treatment efficacy is understandable. Researchers were deterred for looking for MMRd OC as it was thought the prevalence of MMRd in OC would be too low as to be clinically meaningful.^17^ Such an assumption could be made due to a lack of high quality MMRd prevalence data in OC. Therefore, a meta‐analysis of MMRd prevelance in OC is needed as to assess the utility of using MMRd as a biomarker for ICPIs in OC  and to help inform any future ICPI treatment trials in OC.

## 2 OBJECTIVE

The aim of our study was to conduct a systematic review of the literature as to identify and appraise the current evidence base regarding the prevalence of MMRd in OC. In addition, we performed a meta‐analysis as to provide composite results. These data could inform the use of ICPIs in OC.

## 3 METHODS

### 3.1 Eligibility criteria, information sources and search strategy

A systematic review of literature, following PRIMSA guidelines, was performed.^18^ Medline, Embase, Cochrane CENTRAL, NHS Health and Technology and Web of Science were searched from their inception to September 2021. Nonelectronic and gray literature were excluded. Search terms included “DNA mismatch repair,” “ovarian neoplasms” and “colorectal neoplasms, hereditary nonpolyposis” with associated Medical Subject Headings (MeSH); this search strategy was devised by a specialist medical librarian. A secondary search was carried out using “mismatch repair,” “ovarian cancer” and Lynch syndrome” as multipurpose search terms. Initial search results were supplemented by citation searching. The search strategy is detailed in Table S1.

### 3.2 Study selection

The protocol for this systematic review was preregistered with the PROSPERO database registration (ref: CRD42020220975). Studies investigating MMRd in both unselected and selected OC populations were included; that is studies that applied universal testing for MMRd in OC and studies in which the population of OC was selected based on a predefined criterion/criteria, for example histotype specific, were included. Interventional studies in which MMRd testing was carried out were also included. Searches were limited to English language, human adults (>18 years old) and female subjects. No restriction was placed on the date of publication. Studies that used immunohistochemistry (IHC), microsatellite instability analysis (MSI), *MLH1* promotor hypermethylation testing, MMR germline mutation analysis and MMR somatic mutation analysis as diagnostic tools were included. Studies were excluded if they sampled less than 50 OCs or concentrated on synchronous ovarian tumors with other primary malignancies. These exclusions were to ensure high quality data^19^ and prevent selection bias for Lynch syndrome respectively. In addition, articles found to have internal inconsistencies, such as different results reported within the study for the same outcome, were also removed.

### 3.3 Data extraction

Titles and abstracts were collated and screened using Rayyan software (https://rayyan.qcri.org/). Screening was done independently by three authors (A.S.A., M.C.D. and T.A.K.), with any discrepancies reviewed by a third party (N.A.J.R.). Studies that were identified as meeting the inclusion criteria underwent full article review and data extraction by two authors (A.S.A. and T.A.K.), with issues resolved through discussion with a senior author (N.A.J.R.). Articles excluded at full article review are detailed in Table S3. A bespoke data collection tool was designed to ensure complete capture of all primary and secondary outcome data points (available on request). This recorded demographic, pathological and clinical data, and the diagnostic method used to estimate the prevalence of MMRd in women with OC.

### 3.4 Assessment of risk of bias

Risk of bias was assessed using R, version 3.3.1 (https://cran.r-project.org) using the package “robvis”.^20^ This package uses the Quality and Applicability of Diagnostic Accuracy Studies (QUADAS 2) tool for bias analysis.^21^ All primary data is available by request.

### 3.5 Statistical analysis

We defined the primary outcome as the proportion of OC with MMRd defined by the author using either MSI, IHC or somatic sequencing. When estimating the proportion of OC with MMRd by MSI we did not include studies where MSI was only conducted on an unrepresentative sample of patients (eg, if MSI analysis was only performed in patients with MMRd by IHC). An equivalent approach was used when estimating the proportion of OC with MMRd by IHC. All analysis was performed using R version 4.1.0 (https://www.R-project.org/) and the package *meta* version 5.0.^22^

Base case analyses used a generalized linear mixed model with logit transform and random intercepts at the study level (see Equations (1) and (2)). In addition, sensitivity analyses were conducted using a fixed effects approach with logit transform (Equations (1) and (3)) and using an inverse variance approach with Freeman‐Tukey double arcsine transforms (see Table S5). Confidence intervals for individual studies were produced by the Clopper‐Pearson method. Heterogeneity across studies was described with the use of an *I* ^2^ score (low: 25%, 50%: moderate, 75% high heterogeneity). Subgroup effects were tested using the *Q*‐statistic (ANOVA *Q* with $\tau^{2}$ estimated separately in each subgroup).

For the patient j in the study i, the outcome $y_{\mathrm{ij}}$ is equal to 1 in the event of a positive test (eg, when MMR IHC shows MMRd) and is equal to 0 in the event of a negative test.

(1) $$\text{logit}\left(E\left[y_{\mathrm{ij}}\right]\right)=\eta_{\mathrm{ij}},\;\;y_{\mathrm{ij}}∼\text{Bernoulli},$$

(2) $$\eta_{\mathrm{ij}}^{\mathrm{RE}}=\beta_{0}^{\mathrm{RE}}+u_{i}^{\mathrm{RE}},\;u_{i}^{\mathrm{RE}}∼N\left(0,\sigma^{2}\right),$$

(3) $$\eta_{\mathrm{ij}}^{\mathrm{FE}}=\beta_{0}^{\mathrm{FE}}.$$

## 4 RESULTS

### 4.1 Study selection

**FIGURE 1:**
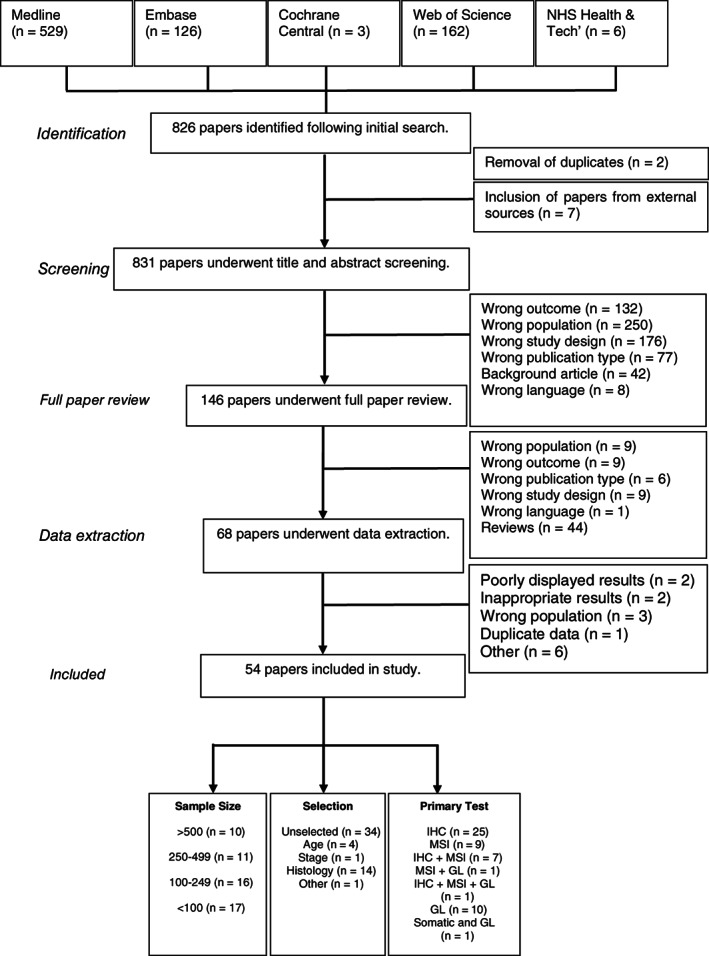
Prisma flow diagram. GL, germline analysis; IHC, immunohistochemistry; MSI, microsatellite instability; Tech, technology

In total 826 articles were identified by the search with an additional seven titles found through citation searching. After abstract screening, 146 articles underwent full article review. During this process a further 78 titles were excluded. Data extraction was performed on 68 articles, however a further 14 articles were excluded due to either not meeting the inclusion criteria, an inability to extract results, internal inconsistencies or duplicate data (see Tables S1 and S2). Therefore 54 articles were included in the meta‐analysis. These data are summarized in Figure 1 and Tables 1 and S6.

**TABLE 1:** Summary of studies

Study feature	Number of studies	Proportion of studies (%)
Country		
North America	26	48.15
Europe	16	29.63
Asia	9	16.67
Mixed continents	2	3.70
Australasia	1	1.85
Selection of patients		
Unselected	34	62.96
Selected	20	37.04
Histology	14	25.93
EC	4	7.40
CCC	4	7.40
EC + CCC	3	5.56
Non‐HGS	1	1.85
Non‐CCC	1	1.85
Non‐serous + non‐mucinous	1	1.85
Age	4	7.40
Stage	1	1.85
Gene	1	1.85
Primary tests for MMRd conducted on unselected patients		
IHC	12	35.29
MSI	10	29.41
GL	7	20.59
IHC + MSI	2	5.88
GL + MSI	1	2.94
GL + somatic	1	2.94
IHC + MSI + somatic	1	2.94

### 4.2 Study characteristics

In total 17 532 OCs were reported in the 54 studies (see Table S4).^23^, ^24^, ^25^, ^26^, ^27^, ^28^, ^29^, ^30^, ^31^, ^32^, ^33^, ^34^, ^35^, ^36^, ^37^, ^38^, ^39^, ^40^, ^41^, ^42^, ^43^, ^44^, ^45^, ^46^, ^47^, ^48^, ^49^, ^50^, ^51^, ^52^, ^53^, ^54^, ^55^, ^56^, ^57^, ^58^, ^59^, ^60^, ^61^, ^62^, ^63^, ^64^, ^65^, ^66^, ^67^, ^68^, ^69^, ^70^, ^71^, ^72^, ^73^, ^74^, ^75^, ^76^ Geographically there was good representation with Europe (n = 16), Australasia (n = 1), North America (n = 26), Asia (n = 9) and multicontinental (n = 2) studies reported. No studies from South America were found. Twenty studies preselected their population on either age (n = 4), stage of disease (n = 1), histology (n = 14) or being path_*BRCA* negative and being <40 years old (n = 1). The mean age of participants was 52 years, however, not all articles (n = 36) reported this parameter. Narrative synthesis was performed for the remaining demographics. In total, 46 articles reported histology breakdown. articles in which all histoypes were reported (n = 33) of, on average, 53% were high grade serous, 18% were endometrioid, 14% were clear cell, 1% were low grade serous and 13% were of other histoypes. Regarding FIGO stage, 49% and 51% were stage I‐II and III‐IV, respectively. Too few articles (n = 3) reported ethnicity to draw a meaningful average. The risk of bias results are displayed in Figure S1, of note no study was free from a degree of bias.

### 4.3 Immunohistochemistry

In total 7086 OCs underwent IHC analysis.^23^, ^24^, ^25^, ^31^, ^32^, ^37^, ^38^, ^39^, ^42^, ^45^, ^46^, ^47^, ^49^, ^50^, ^53^, ^54^, ^55^, ^56^, ^58^, ^60^, ^61^, ^66^, ^72^, ^75^, ^76^ The average age of MMRd OC by IHC was 48 years old; these data come from 13 studies.^24^, ^32^, ^33^, ^36^, ^37^, ^39^, ^43^, ^45^, ^49^, ^61^, ^66^, ^75^, ^76^ This compares to an average age of 61 years in those whose OCs' were MMR proficient. Most studies (n = 23) used tissue microarrays (TMA) for analysis^23^, ^24^, ^25^, ^31^, ^32^, ^37^, ^38^, ^39^, ^42^, ^45^, ^46^, ^47^, ^49^, ^50^, ^53^, ^54^, ^55^, ^56^, ^58^, ^60^, ^61^, ^66^, ^72^, ^75^, ^76^ or a combination of TMA and whole slides.^41^, ^43^ Only seven reported solely using whole slides in their analysis.^26^, ^29^, ^33^, ^34^, ^36^, ^64^, ^71^ In two studies it was not clear if they used TMAs or whole slides.^50^, ^75^

**FIGURE 2:**
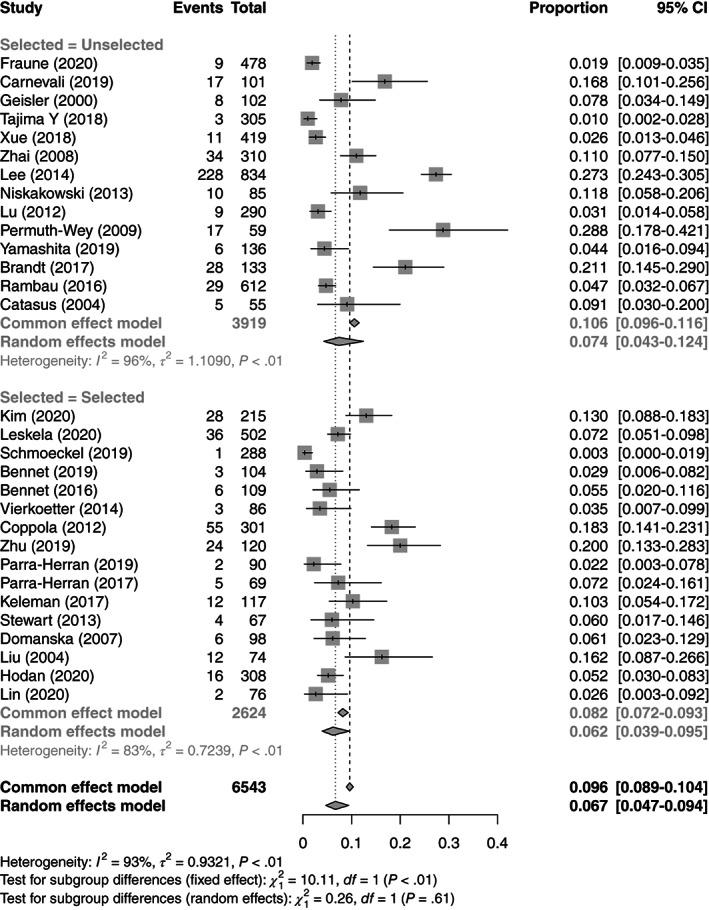
Forest plot for meta‐analysis of test positivity rate for IHC

Thirty studies were included in the IHC meta‐analysis as those studies that only performed IHC as a secondary test were excluded.^38^, ^49^, ^50^ There was a wide variation in the rate of MMRd by IHC, ranging from 0.3% to 29% (see Figure S2). Overall, the estimated rate of OC that demonstrate MMRd by IHC was 6.7% (95% CI: 4.7%‐9.4%). Interestingly, the rates of MMRd by IHC was higher in unselected populations than selected at 7.4% vs 6.2%, respectively. These data are summarized in Figure 2. There was a significant degree of heterogeneity between studies (*I* ^2^ = 93%), which was not adequately explained by subgrouping according to selection into the study (*Q* = 0.26, *df* = 1, *P* = .61).

There were 29 studies that gave information on the specific protein loss; however, 10 of these studies did not test for all four proteins.^25^, ^29^, ^37^, ^38^, ^41^, ^47^, ^50^, ^56^, ^71^, ^72^ Furthermore, two studies did not indicate if loss was isolated or in a dimeric pair.^39^, ^60^ Of note Zhu et al^53^ reported isolated loss of MLH1 and MSH2 which is unusual given the literature on the ability to use a two‐antibody screen in MMRd using PMS2 and MSH6.^77^, ^78^ In addition, Xue et al reported isolated MLH1 loss, however it was not described as absent but as “low expression.”^34^ Therefore 19 studies had sufficient information to describe MMR protein expression in OC.^23^, ^24^, ^26^, ^31^, ^32^, ^33^, ^34^, ^36^, ^42^, ^45^, ^49^, ^53^, ^54^, ^58^, ^61^, ^64^, ^66^, ^75^, ^76^ These studies represent 3987 OCs of which 3986 had IHC testing of which 215 (5.4%) had MMRd. Of these 9 (4%) were reported as isolated MLH1, 82 (38%) had MLH1/PMS2 loss, 2 (1%) had isolated MSH2, 54 (25%) had isolated MSH6 loss and 68 (32%) had MSH2/MSH6 loss. Reflex *MLH1* promotor hypermethylation data was reported in nine studies.^23^, ^24^, ^25^, ^26^, ^32^, ^33^, ^71^, ^75^, ^76^ Of the 53 OCs with MLH1 or MLH1/PMS2 loss 40 (75%) were found to be hypermethylated. Eighteen studies provided information on the FIGO stage in their MMRd IHC cohort.^23^, ^24^, ^31^, ^32^, ^33^, ^34^, ^36^, ^37^, ^39^, ^45^, ^47^, ^49^, ^53^, ^55^, ^60^, ^61^, ^71^, ^76^ In total, 71 (38%) were stage I‐II and 118 (62%) were stage III‐IV. Regarding histotype, 10 unselected studies reported histological data.^25^, ^33^, ^34^, ^42^, ^45^, ^47^, ^49^, ^55^, ^61^, ^71^ MMRd was reported in endometrioid, clear cell, high grade serous, low grade serous and other histologies at 57%, 15%, 12%, 1% and 15%, respectively. We estimate that on average 46.7% (95% CI: 28.8‐65.4) of OCs showing MMRd by IHC had a germline path_*MMR* variant identified. If those not undergoing germline analysis are removed, this becomes 60.5% (95% CI: 39.5‐78.3). This is based on data from nine studies.^23^, ^24^, ^26^, ^32^, ^33^, ^36^, ^39^, ^49^, ^75^ However, many of these studies which were restricted by histology to either clear cell only OCs (n = 2), nonhigh grade serous cancers (n = 2) or endometrioid OC (n = 1).^22^, ^24^, ^30^, ^31^, ^34^, ^37^, ^47^, ^73^

### 4.4 Microsatellite instability analysis

**FIGURE 3:**
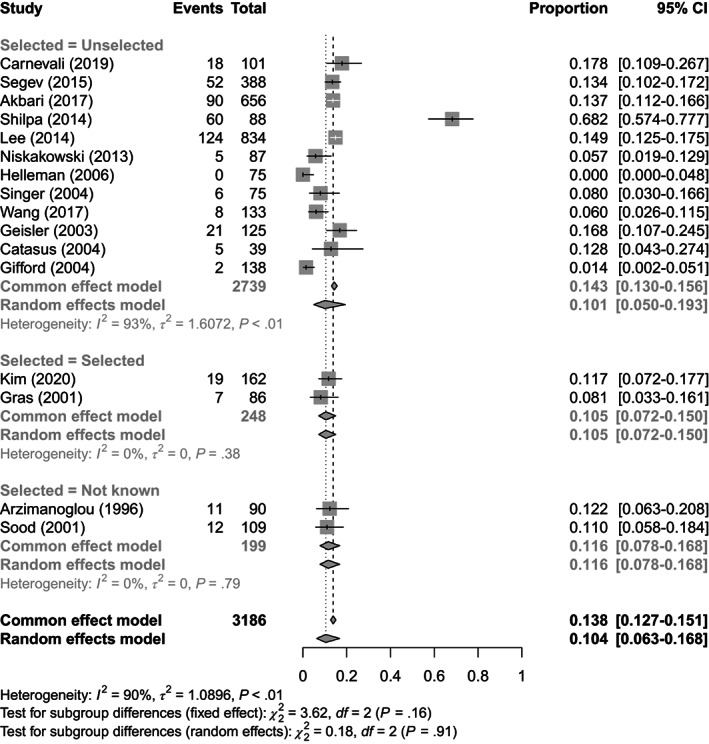
Forest plot for meta‐analysis of test positivity rate for MSI

In total 5472 OCs underwent MSI analysis in 25 studies.^23^, ^25^, ^26^, ^28^, ^31^, ^33^, ^34^, ^35^, ^38^, ^41^, ^42^, ^46^, ^48^, ^49^, ^50^, ^51^, ^55^, ^59^, ^66^, ^67^, ^70^, ^71^, ^73^, ^74^, ^76^ The average age of women with Microsatellite instability high (MSI‐H) OC was 40 years old; these data come from seven studies.^28^, ^33^, ^35^, ^51^, ^59^, ^66^, ^73^ In total 16 studies were included in the MSI meta‐analysis as MSI was used as the primary tumor based test.^23^, ^26^, ^28^, ^35^, ^38^, ^41^, ^42^, ^48^, ^50^, ^51^, ^59^, ^67^, ^70^, ^71^, ^73^, ^74^ These studies reported a significant range of test positivity rates for MSI, from 0% to 68%. Overall, an estimated 10.4% (95% CI: 6.3‐16.8) of OCs demonstrate MMRd by MSI analysis. There was a significant degree of heterogeneity between studies (*I* ^2^ = 90%), which is not adequately explained by subgrouping (*Q* = 0.18, *df* = 2, *P* = .91). When the study by Shilpa et al^38^ was excluded, the $I^{2}$ dropped to 54% and the estimated test positivity rate was 9.4%. Once more, preselection of sample populations had minimal effect on the proportion of MMRd OC. These data are summarized in Figures 3 and S3. Regarding stage and histotype, insufficient studies reported these outcomes limiting any meaningful synthesis.^21^, ^24^, ^33^ We estimate that on average 34.0% (95% CI: 5.9‐81.0) of OCs showing MSI had a germline path_MMR variant identified.

### 4.5 Combined immunohistochemistry and microsatellite analysis

**FIGURE 4:**
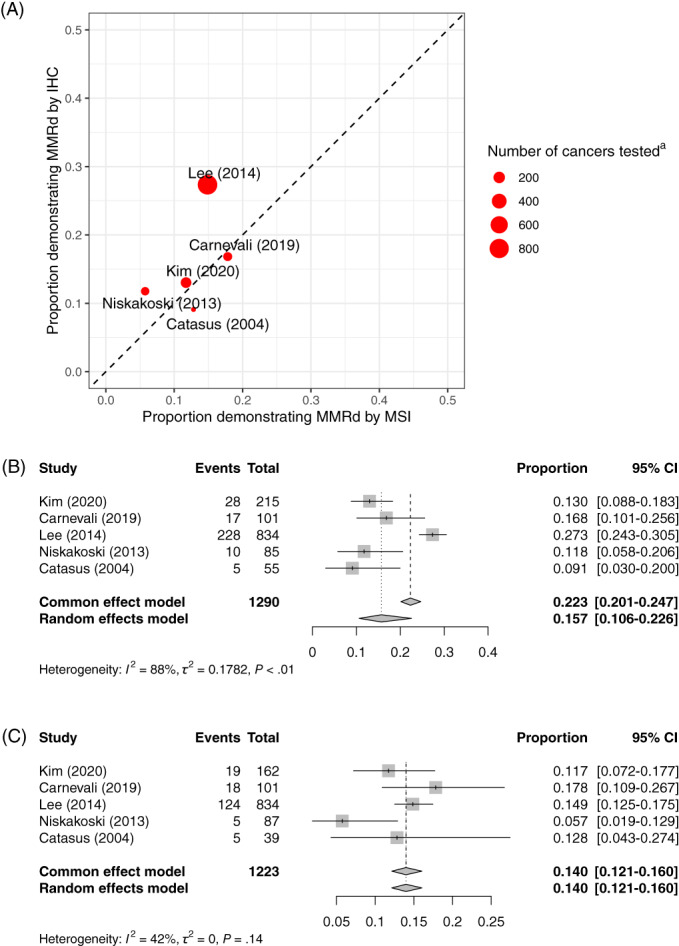
Combined IHC and MSI analysis. (A) Studies conducting MSI and IHC. (B) Forest plot for meta‐analysis of test positivity rate for IHC restricting to studies where unselected MSI was also conducted. (C) Forest plot for meta‐analysis of test positivity rate for MSI restricting to studies where unselected IHC was also conducted. ^a^This is the number of ovarian cancers tested by MSI or IHC (whichever is lower) [Color figure can be viewed at wileyonlinelibrary.com]

A small number of studies conducted IHC and MSI on all OCs (or an unselected subsample).^23^, ^26^, ^41^, ^42^, ^71^ Of note, most of these studies were unselected.^26^, ^41^, ^42^, ^71^ These studies generally show similar test positivity rates, although the study by Lee et al^41^ shows considerably more MMRd by IHC than MSI. Our study specifically found that of 270 discordant cases, 83 were MSI‐H with no loss of expression, while 187 were microsatellite stable (MSS) with loss of IHC expression. In studies with concurrent MSI and IHC, the proportions of MMRd OC were higher than in those studies in which MMRd was defined by one modality. Specifically, the proportion of MSI MMRd OC was 14% vs 10% and the proportion of IHC MMRd in OC was 15.7% vs 6.7%. These data are summarized in Figure 4.

### 4.6 Somatic analysis

Due to the limited number data for somatic analysis, a descriptive synthesis was performed. Four studies conducted somatic MMR mutation analysis.^24^, ^26^, ^27^, ^33^ Carnevali et al^26^ identified 3 (3.0%) OCs with somatic path_*MMR* out of 101 OC evaluated. Sugino et al^27^ identified 11 (5.3%) OCs with somatic path_*MMR* out of 207 cancers evaluated. Tajima et al^33^ identified 2 OCs with somatic path_*MMR* out of 3 OCs evaluated. Leskela et al^24^ conducted somatic MMR mutation analysis on 17 ovarian cancers but results were not reported.

### 4.7 Germline analysis

**FIGURE 5:**
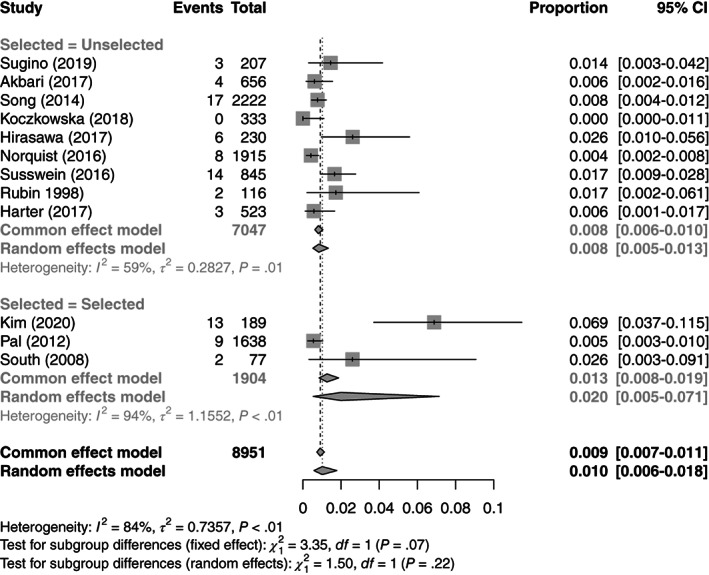
Meta‐analysis of the prevalence of germline path_MMR in studies including an unselected population of ovarian cancers and conducting (near‐) universal germline testing

In total 10 826 OCs in 21 studies underwent some form of germline analysis^23^, ^24^, ^26^, ^27^, ^30^, ^32^, ^33^, ^35^, ^36^, ^39^, ^40^, ^44^, ^49^, ^52^, ^57^, ^62^, ^63^, ^65^, ^68^, ^69^, ^75^; of these, nine preselected their population.^23^, ^24^, ^30^, ^32^, ^36^, ^39^, ^44^, ^65^, ^75^ These studies originated from Europe (n = 6), North America (n = 11), Asia (n = 3) and multicontinental (n = 1). In total 9114 underwent germline analysis in which 122 (1.3%) germline path_*MMR* were identified of which 19 (16%) were path_*MLH1*, 37 (30%) were path_*MSH2*, 52 (43%) were path_*MSH6* and 14 (11%) were path_*PMS2*. Nine studies, representing 4993 OCs, reported variants of unknown significance.^23^, ^33^, ^39^, ^44^, ^52^, ^62^, ^65^, ^68^, ^75^ In total 44 (0.9%) variants of unknown significance were found. A meta‐analysis of studies with near complete germline analysis in an unselected population is displayed in Figure 5. Among unselected OC cases the prevalence of germline path_*MMR* was 0.83% (95% CI: 0.52%‐1.3%). Considering studies in which MSI and/or IHC were conducted and where germline testing was not universal (ie, a situation more like current clinical practice with colorectal and endometrial cancer), we see a yield of germline path_*MMR* among those tested as shown in Figure S4. In these studies, there were generally not many cancers subjected to germline mutation testing, but around half of those tested were found to have germline path_*MMR*.

Ten studies reported that those who had a germline path_*MMR* also had a significant family history.^23^, ^33^, ^35^, ^36^, ^40^, ^44^, ^49^, ^57^, ^65^, ^68^ The percentage of germline path_*MMR* ranged from 0% to 100% with an average of 50%. Twelve studies reported the FIGO stage of OCs with a germline path_MMR.^23^, ^24^, ^27^, ^32^, ^33^, ^35^, ^36^, ^39^, ^49^, ^52^, ^57^, ^62^ Of note, out of the 77 OCs with stage information and a germline path_*MMR*, 62 (81%) were FIGO stage I‐II. Eighteen studies reported the histotype of OCs with a germline path_*MMR*.^23^, ^24^, ^26^, ^27^, ^30^, ^32^, ^33^, ^35^, ^36^, ^39^, ^40^, ^44^, ^49^, ^52^, ^57^, ^62^, ^69^, ^75^ These articles included 268 high grade serous, 1629 endometrioid, 1097 clear cell and 1300 other histotypes. Out of 137 OCs with a germline path_MMR, 79 (58%) were endometroid 21 (15%) were high grade serous, 23 (17%) were clear cell and 14 (10%) were found in another histotype. In unselected studies with complete germline testing,^27^, ^35^, ^40^, ^52^, ^57^, ^62^, ^69^ 2% of endometrioid, 0.4% high grade serous and 1% of clear cell OCs had a germline path_MMR.

### 4.8 Sensitivity analysis

Results of meta‐analyses using the fixed effects model are shown in Figures 2, 3, 4 as “Common effects.” For IHC and MSI these tend to show higher average prevalence of MMRd but given the high degree of heterogeneity it is unlikely that the fixed effects model is appropriate.

The results of meta‐analyses using the inverse variance approach and Freeman‐Tukey double arcsine transforms are shown in Table S2. These show minimal differences with the generalized linear modeling approach.

## 5 COMMENT

### 5.1 Main findings

To the authors' knowledge, we present the most comprehensive review of MMRd in OC. In total, 54 studies were included which detailed MMRd analysis in 17 532 OCs. These data indicate 7% or 10% of OCs are MMRd by IHC or MSI, respectively, although studies where both techniques are used do not suggest that one technique is superior. These data support the existing literature that both IHC and MSI can be used to define MMRd in OC.^79^ This is clinically significant as these cancers would potentially be amenable to ICPIs; a treatment that has been shown be highly effective in solid cancers with MMRd.^10^ Given the poor survival seen in OC, being able to target effective treatments is a clinical priority. In addition, these data would suggest around 1% to 5% of women have Lynch syndrome; although of note, those studies with universal germline testing estimate the prevalence of Lynch syndrome to be closer to 1%. Finding these women is important as they are at risk of synchronous and metachronous cancers; a risk the vast majority are unaware of.^80^ Once diagnosed, those with Lynch syndrome can be enrolled in risk reducing strategies such as routine colonoscopy and aspirin prophylaxis.^81^ Furthermore, cascade testing of the index case's relatives identifies, on average, a further three Lynch syndrome carriers.^82^ These individuals can also benefit from risk reducing strategies that could prevent them from ever developing cancer.^83^ Our data would suggest that 47% of those women found to have MMRd OC by either MSI or IHC went on to be diagnosed with Lynch syndrome. This high proportion could have implication for consenting; currently tumor testing does not require pretesting consent,^84^ however given this high conversion rate clinicians may wish to mention Lynch syndrome testing before tumor analysis.

Of note, our data would, at first look, suggest that germline path_*MSH6* carriers are at the most risk of OC, given it is the most common gene affected in our OC cohort. However, *MSH6* is the gene most commonly affected in those with Lynch syndrome and therefore, the higher proportion of germline path_*MSH6* in OC simply reflects its prevalence in those with Lynch syndrome.^85^ This is demonstrated in population data in which, out of 50 703 healthy individuals tested for germline path_MMR, 9, 13, 23 and 36 had path_*MLH1*, path_*MSH2*, path_*MSH6* and path_*PMS2*, respectively.^86^ The low number of path_*PMS2* in our data can be explained by the weaker association between this gene and OC when compared to other Lynch syndrome causative gene loci.^87^

Our data would suggest the use of MSI or IHC leads to a similar rate of MMRd detection. The use of IHC does give additional information, namely the specific protein that is deficient, which can be useful in the interpretation of variant analysis. Furthermore, if maximizing MMRd yield is the priority, our data would not support the preselection of testing populations. It would seem MMRd is seen in all histotypes of OC, however, is common in endometrioid and rare in low grade serous cancers. Interestingly, the preselection of populations on clinical criteria, such as age or family history, did not significantly improve the yield of MMRd OCs and may miss a significant number of OCs that could be amenable to ICPIs. Therefore, population preselection does not seem beneficial.

### 5.2 Strengths and limitations

Our study has several key strengths. First, our search strategy was designed to capture all the relevant literature. This means our conclusions are based on the results of 54 studies and 17 532 OCs. The systematic review and meta‐analysis followed PRISMA guidelines.^18^ Both article screening, risk of bias assessment and data extraction was performed by at least two independent reviewers. Meta‐analyses were conducted using appropriate methods for the type of data collected, and multiple sensitivity analyses were conducted to ensure the robustness of results.

However, there are certain limitations in our review approach and in the body of evidence identified. We took results as reported and did not seek to review original study data. We note a high degree of risk of bias within studies. Studies also used mixed methods such as TMAs vs whole slide analysis and different MSI assays. We have not attempted to identify publication bias (eg, smaller studies finding very low rates of MMRd in OC may not be published, or the rates of MMRd may not be reported when other characteristics are reported). In addition, incomplete testing led to incomplete data sets which were difficult to incorporate into our analysis. Furthermore, many studies were retrospective and used historical cohorts. The lack of a “gold standard” test for MMRd in OC makes accuracy estimates difficult. Our studies span a wide time frame (1998‐2020), during which diagnostic technologies have changed; this could impact on prevalence estimates. However, this would minimally affect IHC which has remained consistent. In addition, because most studies that use MSI, and sequencing technology are from 2015 or later, the impact should be limited. Finally, the data has a bias towards Western Caucasian populations which makes the generalizability to other populations less robust. This could have been further compounded by our pragmatic decision to only include studies written in English. Germline testing has implications for both the index case and their family. Due to the implications of a Lynch syndrome diagnosis in insurance‐based health care systems, women may have declined to take part in prospective studies in insurance‐based health care populations; this could potentially limit the validity of our germline results.^88^ These issues are reflected in our wide confidence intervals around our estimated prevalence for MMRd in OC and in the high $I^{2}$ scores. Where possible we tried to mitigate this by performing subgroup analysis. This heterogeneity and the need to include low quality studies limits the strength of our conclusions.

### 5.3 Comparison with existing literature

Regarding those with a germline MMRd, our data found OCs in this population seem to be diagnosed at stage I or II disease. This has been reported before in Lynch syndrome populations.^89^ This is an interesting finding as most OCs are diagnosed at a more advanced stage due to a diagnostic difficultly because of no screening test^90^ and no pathognomonic symptom.^91^ This finding could speak to Lynch syndrome associated OCs having a different biology. Indeed, it is known these cancers have an immunogenic profile, which limits their ability to metastasize.^92^ These findings warrant more exploration in future studies.

Our data cannot speak to the effectiveness of ICPIs in MMRd OCs as this was not our aim. However, it is known these agents are highly effective in solid cancers with MMRd.^93^ Their clinical utility in MMRd cancers is such that they were the first chemotherapeutic to receive FDA approval based on a molecular feature within a cancer as opposed to the anatomical origin of the cancer.^94^ Yet these agents have been used in OC with limited success.^95^ What is known is that ICPIs have a low toxicity profile in women with OC.^96^ The JAVELIN ovarian 100 study explored the use of avelumab both in combination with standard chemotherapy and as a maintenance agent.^16^ The study was stopped early as it failed to show any benefit for the use of avelumab in either arm; indeed, there was a suggestion of a detrimental effect. The NINJA study also compared an ICPI with standard chemotherapy; again, the authors failed to demonstrate an improvement in overall or progression free survival.^97^ However, neither of these studies selected OC with MMRd and therefore it is not known if ICPIs would be of benefit in a MMRd OC population. The JAVELIN Ovarian 200 study also explored the potential benefit of ICPI use in OC and once more failed to demonstrate a survival benefit.^98^ However, the authors did suggest that the use of biomarkers (CD8 and PD‐L1 expression) to select OC as candidates for ICPIs may aid in finding women who would gain a survival benefit from ICPI use; however, the study was underpowered to fully explore this. MMRd cancers are known to express higher levels of PD‐L1 and CD8.^92^ Our data shows that MMRd testing in OC is possible. What is more, it is more prevalent than previously thought. Therefore, trials exploring checkpoint inhibition in MMRd OC should be considered.

### 5.4 Conclusions and implications

In summary, we present the most comprehensive systematic review and meta‐analysis exploring MMRd in OC. We found that a significant minority (up to 16%) of OC displays MMRd and therefore could be amenable to ICPIs. However, the current literature base is of limited quality and therefore high‐quality prospective studies exploring MMRd in OC with the use of multimodal testing are required. In addition, trials looking at the efficacy of check point inhibition in MMRd OC are needed.

## AUTHOR CONTRIBUTIONS

*Conception and design*: Neil A. J. Ryan, Claire Newton, D. Gareth Evans and Emma J. Crosbie. *Financial support*: None. *Collection and assembly of data*: Neil A. J. Ryan, Amit Atwal, Thomas Krum and Marcus Cabrera Dandy. *Data analysis and interpretation*: Neil A. J. Ryan and Tristan Snowsill. *Article writing*: Neil A. J. Ryan and Amit Atwal. *Final approval of article*: All authors. The work reported in the article has been performed by the authors, unless clearly specified in the text.

## FUNDING INFORMATION

No specific funding was used for our study. Emma J. Crosbie is supported by the NIHR Manchester Biomedical Research Centre (IS‐BRC‐1215‐20007) and an NIHR Advanced Fellowship (NIHR300650). D Gareth Evans is an NIHR Senior Investigator (NF‐SI‐0513‐10076).

## CONFLICT OF INTEREST

The authors declare no conflicts of interest.

## ETHICS STATEMENT

All studies were approved by the respective institutional review boards and conducted with appropriate ethical criteria in each country and in accordance with the Declaration of Helsinki.

## Supporting information


**Appendix S1** Supporting Information.

## Data Availability

All data contained here within this article is available on request from the corresponding author.
