# Supplementary material for: The prevalence of mismatch repair deficiency in ovarian cancer: A systematic review and meta‐analysis
Source: Int J Cancer. 2022 Jul 6;151(9):1626–39. doi: 10.1002/ijc.34165 (PMC9539584; doi:10.1002/ijc.34165)
Supplement: Supplementary file 1 — Appendix S1 Supporting Information. [file IJC-151-1626-s001.pdf]

Supplementary Materials for
The prevalence of mismatch repair deficiency in ovarian cancer: a systematic review and meta-analysis

Table S1 Search strategy 2
Table S2: Studies excluded at full manuscript review 2
Table S3 Studies excluded during data extraction...4
Table S4 Included studies characteristics 5
Table S5 Meta-analysis of test sensitivity using Freeman-Tukey arcsine transformation. 31
Table S6 PRISMA check list 31

Figure S1 Risk of bias assessment using Robvis[1] 35
Figure S2 Proportion of ovarian cancer with mismatch repair deficiency by immunohistochemistry as reported by individual studies 36
Figure S3 Proportion of ovarian cancer with mismatch repair deficiency by microsatellite instability analysis as reported by individual studies 37
Figure S4 Proportion of ovarian cancer with a germline path_MMR in Considering studies in which MSI and/or IHC were conducted 38

Table S1 Search strategy

Column number	Term	No. of results
**1**	Mismatch repair.mp. or DNA Mismatch Repair/	11320
**2**	ovarian cancer.mp. or Ovarian Neoplasms/	97681
**3**	1 and 2	411
**4**	Lynch syndrome.mp. or Colorectal Neoplasms, Hereditary Nonpolyposis/	5833
**5**	4 or 1	14428
**6**	3 and 5	752

Table S2: Studies excluded at full manuscript review

Title	Author	Study year	Reason for exclusion
Molecular Profiling Reveals Common and Specific Development Processes in Different Types of Gynecologic Cancers	Guo *et al.*	2020	Wrong outcome. Did not report on any MMR genes
Improving attendance to genetic counselling services for gynaecological oncology patients.	Pokharel *et al.*	2018	Not assessing prevalence of MMR deficiency in OC
Evaluation of a 27-gene inherited cancer panel across 630 consecutive patients referred for testing in a clinical diagnostic laboratory.	Gardner *et al.*	2018	Wrong outcome. Reporting experience using multi-gene panel testing.
Hypermethylation of mismatch repair gene hMSH2 associates with platinum-resistant disease in epithelial ovarian cancer.	Tian *et al.*	2019	Wrong outcome. Assessing hypermethylation of hMSH2
PMS2 germline mutation c.943C>T (p.Arg315*)-induced Lynch syndrome-associated ovarian cancer.	Guo *et al.*	2019	Less than 50 participants
Genetic polymorphisms in hMSH2 and hMLH1 genes are associated with prognosis in epithelial ovarian cancer patients.	Si *et al.*	2019	Wrong outcome. Assessing polymorphisms.
Contribution of Massive Parallel Sequencing to Diagnosis of Hereditary Ovarian Cancer in the Czech Republic.	Soukupová *et al.*	2019	Wrong language
The founder mutation MSH2*1906G-->C is an important cause of hereditary nonpolyposis colorectal cancer in the Ashkenazi Jewish population.	Foulkes *et al*	2002	Identification of founder mutation in population which already are known to have LS

Prognostic values of DNA mismatch repair genes in ovarian cancer patients treated with platinum-based chemotherapy.	Zhao *et al.*	2017	Wrong outcome. Assessing prognostic value of MMR genes not prevalence
A Cross-Cancer Genetic Association Analysis of the DNA Repair and DNA Damage Signaling Pathways for Lung, Ovary, Prostate, Breast, and Colorectal Cancer.	Scarbrough *et al.*	2016	Wrong outcome. Investigating SNPs
Family history and BRCA1/BRCA2 status among Japanese ovarian cancer patients and occult cancer in a BRCA1 mutant case.	Hirasawa *et al.*	2014	Wrong outcome. Diagnosis of LS provided on basis of FH
Expression and promoter methylation status of mismatch repair gene hMLH1 and hMSH2 in epithelial ovarian cancer.	Zhang *et al.*	2008	Wrong outcome. Assessing methylation status of hMLH1 and hMSH2 and its relationship with gene expression
Expression of factors involved in regulation of DNA mismatch repair- and apoptosis pathways in ovarian cancer patients.	Materna *et al.*	2007	Less than 50 participants
Are DNA mismatch repair deficiencies responsible for accumulation of genetic alterations in epithelial ovarian cancers?.	Suzuki *et al.*	2001	No MMR genes reported
Clinical outcomes of a genomic screening program for actionable genetic conditions.	Buchanan *et al.*	2020	Wrong study design. Assessing impact of genomic screening on risk management and early detection. Population already had LS
Genetic testing for lynch syndrome, an inherited cancer of the bowel, endometrium, and ovary.	Strafford *et al.*	2012	Review
Microsatellite analysis of sporadic and hereditary gynaecological cancer in routine diagnostics.	Libera *et al.*	2017	Less than 50 participants
SEOM clinical guidelines for hereditary cancer.	Begoña Graña *et al.*	2011	Review
Recent progress in the diagnosis and treatment of ovarian cancer.	Lejovac *et al.*	2011	Review

[Lynch syndrome: towards a multidisciplinary management of tumour screening]	Bats *et al.*	2011	Review and wrong language
Mismatch repair gene expression defects contribute to microsatellite instability in ovarian carcinoma.	Pal *et al.*	2004	Wrong publication type
The silence of the genes: matching mismatch repair defects with tumors.	Boland *et al.*	2003	Review
Ovarian tumors display persistent microsatellite instability caused by mutation in the mismatch repair gene hMSH-2.	Orth *et al.*	1994	Wrong population. Investigation conducted on cancer cell lines

Table S3 Studies excluded during data extraction

**Author**	**DOI or PMID**	**Reason for exclusion**
Lee 2014	10.1089/gtmb.2013.0393	Wrong population (included borderline tumours)
Aysal 2012	10.1097/PAS.0b013e31823bc434	Wrong population (<50 pure OC)
La Duca 2012	10.1038/s41436-019-0633-8	Wrong outcome (unable to differentiate ovarian cancer results from cohort)
Rosen 2006	10.1038/modpathol.3800672	Wrong population (unable to differentiate 2 synchronic endometrial endometrioid carcinomas included in study - both of which were MMR-D)
Lu 2019	10.1001/jamaoncol.2018.2956	Wrong outcome (Did not use any of IHC, MSI, Methylation, Somatic or germline analysis)
Song 2006	https://doi-org.bris.idm.oclc.org/10.1093/carcin/bgl089	Wrong study design
Stasikowska-Kanicka 2009	PMID: 20072951	Wrong population (includes benign tumours)

Table S4 Included studies characteristics

Author	Study year	Country	Type of study	Design of study	Population	Selection	Number of participants	Initial tumour screen	Proportion of positive IHC	Proportion of positive MSI	Proportion of negative methylation after positive tumour triage	Proportion of positive germline samples after positive tumour-triage	Comments
**Akbari et al**	2017	USA & Canada	Retrospective	Cross-sectional	Three population-based studies of OEC. 1) The Familial Ovarian Tumour Study (FOTS) in Toronto, 2) the Tampa Bay Ovarian Cancer Study (TBOCS), 3) the North Carolina Ovarian Cancer Study (NCOCS).	U/S	656	MSI + GL	N/A	0.14	N/A	N/A	Universal GL testing conducted.

**Arzimanoglou et al**	1996	USA	Prospective	Cross-sectional	OC specimens from surgery and venous blood collected within a day of surgery.	U/S	90	MSI	N/A	0.12	N/A	N/A	MSI tested using 3 loci panel.
**Bennet et al**	2016	USA	Retrospective	Cross-sectional	Surgical pathology archives at Massachusetts General Hospital and Stanford Medical centre.	CCC	109	IHC	0.06	N/A	1	1	GL testing conducted on 4 of 6 MMR-D tumours.
**Bennet et al**	2019	USA	Retrospective	Cross-sectional	Surgical pathology archives at Massachusetts General Hospital, Ospedale Sacro Cuore-Don Calabria, and Lahey Hospital and Medical Centre.	EC	104	IHC	0.03	N/A	N/A	1	GL testing completed on 2 of 3 MMR-D tumours.

**Brandt et al**	2017	Switzerland	Retrospective	Cross-sectional	Cancer samples from the archive from the Institute of Surgical Pathology, University Hospital Zurich.	U/S	144	IHC	0.21	N/A	N/A	N/A	Only MLH1 & MSH2 tested. Inconsistencies in reported data. No gene breakdown provided.
**Carnevali et al**	2019	Italy	Retrospective	Cross-sectional	Patients with histologically proven OC who were referred to Cancer Genetic Service of ASST Settelaghi Hospital.	Patients referred to a genetics service	207	IHC + MSI	0.17	0.18	0	0.75	GL analysis also done on atypical OC tumours
**Catasus et al**	2004	Spain	Retrospective	Cross-sectional	Clinicopathological records of patients with OC.	U/S	55	IHC	0.09	0.13	0.6	N/A	Only MLH1 tested. MSI done in 39 cases.

**Coppola et al**	2012	USA & Canada	Retrospective	Cross-sectional	Three population-based studies of OEC. 1) The Familial Ovarian Tumour Study (FOTS) in Toronto, 2) the Tampa Bay Ovarian Cancer Study (TBOCS), 3) the North Carolina Ovarian Cancer Study (NCOCS).	Age > 20	301	IHC	0.18	N/A	N/A	N/A	Full section cases excluded as demographic & clinical analysis not completed.
**Domanska et al**	2007	Sweden	Retrospective	Cross-sectional	Histopathology reports of OEC.	Age < 40	98	IHC	0.06	0.83	N/A	N/A	MSI done on panel of 4 markers.

**Fraune et al**	2020	Germany	Retrospective	Cross-sectional	Tissue microarray samples of OC diagnosed at the University Medical Centre Hamburg-Eppendorf.	U/S	478	IHC + MSI	0.02	0.7	0.33	N/A	15 cases classed MMR intact as 3/4 proteins were intact and readable.
**Fuseya et al**	2012	Japan	Retrospective	Cross-sectional	Histopathology archive of OEC at Shinshu University Hospital.	U/S	64	IHC + MSI	N/A	0.26	N/A	N/A	IHC data not provided. MSH6 & PMS2 not tested; MSI completed on 47 patients.
**Geisler et al**	2000	USA	Prospective	Cross-sectional	Patients with OEC whose initial surgery was performed by one gynae-oncologist (H.E.G.).	U/S	102	IHC	0.08	N/A	N/A	N/A	Only tested MSH2.

**Geisler et al**	2003	USA	Retrospective	Cross-sectional	Snap-frozen OC samples available for reverse transcriptase-polymerase chain reaction.	U/S	125	MSI	N/A	0.17	N/A	N/A	Universal methylation testing of MSH2 and MLH1 done, 18 were methylated.
**Gifford et al**	2004	UK	Retrospective	Cross-sectional	Patients with OEC who enrolled in the SCOTROC1 phase III randomised trial.	U/S	138	MSI	N/A	0.01	N/A	N/A	Universal methylation testing done. 16 found methylated.
**Gras et al**	2001	Spain	Retrospective	Cross-sectional	Patients with ovarian tumours from surgical pathology files at Hospital Santa Creu I Sant Pau	EC + CCC	86	MSI	N/A	0.08	N/A	N/A	Methylation testing done on 6 MSI-H & 21 MSS tumours. 6 were methylated.

**Harter et al**	2017	Germany	Prospective	Cross-sectional	Patients with primary diagnosis or platinum-sensitive relapse of invasive OEC from 20 cancer centres.	U/S	523	GL	N/A	N/A	N/A	N/A	3 of 523 had GL path_MMR.
**Helleman et al**	2006	Netherlands	Prospective	Cross-sectional	Tissue of OC patients collected at the Erasmus MC in Rotterdam	U/S	75	MSI	N/A	0	N/A	N/A	Panel of 4 MSI markers used. All tumours received methylation testing.
**Hirasawa et al**	2017	Japan	Retrospective	Cross-sectional	Samples from patients with OC that had been stored in Keio Women's Health Biobank, in Tokyo Japan.	U/S	230	GL	N/A	N/A	N/A	N/A	6 of 230 found to be GL path_MMR.

**Hodan et al**	2020	USA	Prospective	Cross-sectional	Cases of primary ovarian, fallopian, and peritoneal cancer between January 2012 and December 2019.	Selected for non-serous and non-mucinous	308	IHC	0.05	N/A	0	0.67	None
**Keleman et al**	2017	USA	Retrospective	Cross-sectional	Medical records and tumour tissue micro arrays from the Alberta Cancer Registry.	EC + CCC	293	IHC	0.1	N/A	N/A	N/A	No MLH1/PMS2 or MSH2/MSH6 mutation provided, All mutations given as individual mutations. IHC Analysis only done on 117 tumours.

**Kim et al**	2020	Canada	Prospective	Cross-sectional	Three Ontario gynae-oncology centres between September 2015 and June 2019.	Non-serous and non-mucinous	215	IHC + MSI + GL	0.13	0.12	0.2	N/A	Analysis includes 30 synchronous endometrial cancers (EC).
**Koczkowska et al**	2018	Poland	Prospective	Cross-sectional	OC patients who were referred to the University Hospital in Gdansk and Red Cross Hospital in Gdynia between 2012 and 2013	U/S	333	GL	N/A	N/A	N/A	N/A	No GL mutations found.

**Lee et al**	2014	USA & Canada	Retrospective	Cross-sectional	Three population-based studies of OEC. 1) The Familial Ovarian Tumour Study (FOTS) in Toronto, 2) the Tampa Bay Ovarian Cancer Study (TBOCS), 3) the North Carolina Ovarian Cancer Study (NCOCS).	U/S	834	IHC + MSI	0.27	0.15	N/A	N/A	PMS2 not tested.

**Leskela et al**	2020	Spain	Prospective	Cohort	Samples from a prospective register of patients who were treated at 28 Spanish Group of Ovarian Cancer Research Centres (GEICO) between 1998 and 2019.	FIGO stage 1-2 only.	502	IHC	0.07	N/A	0.19	0.35	GL analysis not completed on all IHC MMR-D tumours.
**Lin et al**	2020	Taiwan	Retrospective	Cross-sectional	Surgical pathology archives of the Department of Pathology and Laboratory Medicine, Taipei Veterans General Hospital.	CCC	76	IHC	0.03	0.5	0	N/A	MSI analysis conducted on MMR-D tumours only.
**Liu et al**	2004	USA	Prospective	Cross-sectional	Not known	EC	74	IHC	0.16	N/A	N/A	N/A	None

**Lu et al**	2012	Canada	Retrospective	Cross-sectional	Histopathology Achieves	U/S	290	IHC	0.03	N/A	N/A	N/A	22 Borderline tumours excluded.
**Malander et al**	2006	Sweden	Retrospective	Cross-sectional	Histopathology archives.	U/S	128	IHC	0.02	0.75	N/A	0.67	MSI conducted in 1 MMR proficient tumour.
**Niskakoski et al**	2013	Finland	Retrospective	Cross-sectional	Histopathology archives from the Helskini University Central Hospital and Jyväskylä Central Hospital.	U/S	87	IHC + MSI	0.12	0.06	N/A	N/A	IHC completed on 85 patients.

**Norquist et al**	2016	USA	Prospective	Cross-sectional	Patients with OC from three sources: 1) patients undergoing primary treatment at the University of Washington Medical Centre, 2) patients who consented for translational research with available DNA from Gynaecological Oncology Group (GOG) protocol 218, and 3) GOG protocol 262.	U/S	1915	GL	N/A	N/A	N/A	N/A	8 of 1915 patients were GL path_MMR.

**Pal et al**	2012	USA & Canada	Retrospective	Cross-sectional	Three population-based studies of OEC. 1) The Familial Ovarian Tumour Study (FOTS) in Toronto, 2) the Tampa Bay Ovarian Cancer Study (TBOCS), 3) the North Carolina Ovarian Cancer Study (NCOCS).	Age > 20	1638	GL	N/A	N/A	N/A	0.01	PMS2 not tested. 255 borderline tumours excluded.

**Parra-Herran et al**	2017	Canada	Retrospective	Cross-sectional	OC patients treated surgically at Sunnybrook Health Sciences Centre between June 2000-December 2013 were retrieved from the Anatomic Pathology database.	EC	69	IHC	0.07	N/A	N/A	N/A	None

**Parra-Herran et al**	2019	Canada	Retrospective	Cross-sectional	OC patients treated surgically at Sunnybrook Health Sciences Centre between June 2000-December 2013 and January 2014-December 2017 were retrieved from the Anatomic Pathology database.	CCC	90	IHC	0.02	N/A	N/A	N/A	None
**Permuth-Wey et al**	2009	USA	Retrospective	Cross-sectional	OEC tumour samples obtained from the Tampa Bay Ovarian Cancer Study	U/S	59	IHC	0.29	N/A	N/A	N/A	PMS2 not tested.

**Rambau et al**	2016	USA	Retrospective	Cohort	Participants drawn from the Alberta Ovarian Tumour Type (AOVT) study, identified through the Alberta Cancer Registry between 1978-2010.	U/S	612	IHC	0.05	N/A	N/A	N/A	None
**Rubin et al**	1998	USA	Retrospective	Cross-sectional	Patients with OEC treated at the University of Pennsylvania between 1994-1996.	U/S	116	GL	N/A	N/A	N/A	N/A	2 of 116 were GL path_MMR
**Schmoeckel et al**	2019	Germany	Retrospective	Cross-sectional	OC diagnosed between 2003-2007 at the Institute of Pathology, Ludwig-Maximilians-University, Munich, Germany.	non-CCC	288	IHC	0	1	N/A	N/A	MSI analysis conducted on the 1 MMR-D tumour.

**Segev et al**	2015	Canada	Retrospective	Cross-sectional	Participants were drawn from the Familial Ovarian Tumour Study (FOTS) in Toronto, where OEC cases were identified from pathology reports submitted to the Onatrio Cancer Registry.	U/S	418	MSI	N/A	0.13	N/A	N/A	MSI conducted on 388 tumours, no reason given for loss to follow up.
**Shilpa et al**	2014	India	Prospective	Cross-sectional	Patients admitted to the department of Gynae Oncology, Kidwai Memorial Institute of Oncology, Bangalore.	U/S	88	IHC + MSI	0.28	0.68	0.53	N/A	MSH6 not tested, methylation conducted on 88 patients and done for MSH2 and PMS2.

**Singer et al**	2004	Switzerland	Retrospective	Cross-sectional	Archives of the Institute of Pathology, University of Basel Switzerland.	U/S	75	MSI	0.83	0.08	N/A	N/A	IHC only done on MSI-H tumours. Only MLH1 & MSH2 tested.
**Song et al**	2014	UK & USA	Retrospective	Case-control	Invasive OEC cases drawn from two case control studies: the population-based SEARCH study from the UK, and the hospital-based Mayo clinic study from the USA.	U/S	2222	GL	N/A	N/A	N/A	N/A	Universal GL testing conducted; 17 tumours were path_MMR.
**Sood et al**	2001	USA	Prospective	Cross-sectional	Patients with OC who underwent surgical exploration and cytoreduction as the initial treatment.	U/K	109	MSI	N/A	0.11	N/A	N/A	Unknown if population was selected or unselected.

**South et al**	2008	USA	Prospective	Cross-sectional	Participants were identified from the Gilda Radner Familial Ovarian Cancer Registry (GRFOCR).	-ve BRCA	77	GL	N/A	N/A	N/A	N/A	2 of 77 tumours were GL path_MMR; PMS2 not tested.
**Stewart et al**	2013	Australia	Retrospective	Cross-sectional	Histopathology files of King Edward Memorial Hospital (1992-2008) and SJOJ Pathology (1999-2008) Perth.	EC	67	IHC	0.06	N/A	N/A	N/A	35 tumours were associated with endometriosis

**Stratton et al**	1999	United Kingdom	Retrospective	Cohort	Women with OEC were invited to participate through the following UK cancer registries: East Anglian, Thames, Trent, Wessex, Oxford, South West, West Midlands, Mersey and Cheshire, Wales, West of Scotland, Scottish and Northern Ireland.	Age < 30 years old	100	GL	N/A	N/A	N/A	N/A	GL testing only completed for MLH1 & MSH2.

**Sugino et al**	2019	Japan	Retrospective	Cohort	Patients with OEC who had undergone initial surgery between July 2006 and September 2017 at Niigata University Medical and Dental Hospital.	U/S	207	Somatic + GL	N/A	N/A	N/A	N/A	Somatic testing found 11 somatic path_MMR in 207 subjects.
**Susswein et al**	2016	USA	Prospective	Cross-sectional	Patients referred for evaluation Next Generation Sequencing between August 2013 and October 2014.	U/S	845	GL	N/A	N/A	N/A	N/A	Inconsistencies recorded in GL data. 14 of 845 were GL path_MMR.

**Tajima et al**	2018	Japan	Retrospective	Cross-sectional	Clinicopathological records of patients with OEC who underwent surgery between April 2005 and September 2014.	U/S	305	IHC	0.01	1	0.5	0	MSI done on 3 MMR-D tumours.
**Vierkoetter et al**	2014	USA	Retrospective	Cross-sectional	Cases at the Queens Medical Centre in Honolulu diagnosed between January 1st 1995– April 12th 2013.	CCC + EOC	86	IHC	0.03	N/A	N/A	0.5	4 synchronous tumours excluded.

**Wang et al**	2017	Canada	Retrospective	Cross-sectional	The OvCaRe gynaecological tissue bank, the CRCHUM Ovarian Cancer Tumour Bank and the Atomical Pathology archives at the Jikei University school of Medicine.	U/S	133	MSI	N/A	0.06	N/A	N/A	None
**Xue et al**	2018	China	Retrospective	Cross-sectional	OEC cases from 2008-2016 were retrieved from the pathology department of Sichuan provincial people's hospital.	U/S	419	IHC + MSI	0.03	0.21	N/A	N/A	MSI conducted on MMR-Low tumours as well.

**Yamashita et al**	201 9	Japan	Retrospect ive	Cross-section al	Patients with OC treated between January 1997 and December 2017 in the Department of Obstetrics and Gynaecology at Shimane University Hospital.	U/S	136	IHC	0.04	1	N/A	N/A	MSI conducted on MMR-D tumours only.
**Zhai et al**	200 8	USA	Retrospect ive	Cross-section al	Patients with primary OEC who had undergone initial surgery at the University of Texas M.D. Anderson Cancer Centre between 1900-2000 for whom tissue samples and medical records were available.	U/S	322	IHC	0.11	N/A	N/A	N/A	Only assessed MSH6. 12 tumours excluded due to loss of cores.

**Zhu et al**	2019	China	Retrospective	Cross-sectional	The archival collections at Fudan University Shanghai Cancer Centre between 2000-2014.	CCC	120	IHC	0.2	N/A	N/A	N/A	Inconsistency reported in tumour staging data.

Table S5 Meta-analysis of test sensitivity using Freeman-Tukey arcsine transformation.

Analysis	Est. (RE)	95% LCL (RE)	95% UCL (RE)	Est. (FE)	95% LCL (FE)	95% UCL (FE)
MMRd per IHC	0.079	0.055	0.106	0.078	0.072	0.085
MMRd per IHC (studies also performing MSI)	0.159	0.100	0.228	0.217	0.195	0.241
MMRd per MSI	0.119	0.064	0.187	0.127	0.115	0.139
MMRd per MSI (studies also performing IHC)	0.126	0.091	0.167	0.136	0.117	0.156
Prevalence of germline path_MMR (universal testing)	0.011	0.005	0.020	0.007	0.005	0.008
Yield of germline path_MMR (targeted testing)	0.650	0.386	0.881	0.645	0.499	0.782

Table S6 PRISMA check list

Section and Topic	Item #	Checklist item	Location where item is reported
**TITLE**			
Title	1	Identify the report as a systematic review.	
**ABSTRACT**			
Abstract	2	See the PRISMA 2020 for Abstracts checklist.	
**INTRODUCTION**			
Rationale	3	Describe the rationale for the review in the context of existing knowledge.	
Objectives	4	Provide an explicit statement of the objective(s) or question(s) the review addresses.	
**METHODS**			
Eligibility criteria	5	Specify the inclusion and exclusion criteria for the review and how studies were grouped for the syntheses.	
Information sources	6	Specify all databases, registers, websites, organisations, reference lists and other sources searched or consulted to identify studies. Specify the date when each source was last searched or consulted.	
Search strategy	7	Present the full search strategies for all databases, registers and websites, including any filters and limits used.	
Selection process	8	Specify the methods used to decide whether a study met the inclusion criteria of the review, including how many reviewers screened each record and each report retrieved, whether they worked independently, and if applicable, details of automation tools used in the process.	
Data collection process	9	Specify the methods used to collect data from reports, including how many reviewers collected data from each report, whether they worked independently, any processes for obtaining or confirming data from study investigators, and if applicable, details of automation tools used	

Section and Topic	Item #	Checklist item	Location where item is reported
		in the process.	
Data items	10a	List and define all outcomes for which data were sought. Specify whether all results that were compatible with each outcome domain in each study were sought (e.g. for all measures, time points, analyses), and if not, the methods used to decide which results to collect.	
	10b	List and define all other variables for which data were sought (e.g. participant and intervention characteristics, funding sources). Describe any assumptions made about any missing or unclear information.	
Study risk of bias assessment	11	Specify the methods used to assess risk of bias in the included studies, including details of the tool(s) used, how many reviewers assessed each study and whether they worked independently, and if applicable, details of automation tools used in the process.	
Effect measures	12	Specify for each outcome the effect measure(s) (e.g. risk ratio, mean difference) used in the synthesis or presentation of results.	
Synthesis methods	13a	Describe the processes used to decide which studies were eligible for each synthesis (e.g. tabulating the study intervention characteristics and comparing against the planned groups for each synthesis (item #5)).	
	13b	Describe any methods required to prepare the data for presentation or synthesis, such as handling of missing summary statistics, or data conversions.	
	13c	Describe any methods used to tabulate or visually display results of individual studies and syntheses.	
	13d	Describe any methods used to synthesize results and provide a rationale for the choice(s). If meta-analysis was performed, describe the model(s), method(s) to identify the presence and extent of statistical heterogeneity, and software package(s) used.	
	13e	Describe any methods used to explore possible causes of heterogeneity among study results (e.g. subgroup analysis, meta-regression).	
	13f	Describe any sensitivity analyses conducted to assess robustness of the synthesized results.	
Reporting bias assessment	14	Describe any methods used to assess risk of bias due to missing results in a synthesis (arising from reporting biases).	
Certainty assessment	15	Describe any methods used to assess certainty (or confidence) in the body of evidence for an outcome.	
**RESULTS**			
Study selection	16a	Describe the results of the search and selection process, from the number of records identified in the search to the number of studies included in the review, ideally using a flow diagram.	
	16b	Cite studies that might appear to meet the inclusion criteria, but which were excluded, and explain why they were excluded.	
Study	17	Cite each included study and present its characteristics.	

Section and Topic	Item #	Checklist item	Location where item is reported
characteristics			
Risk of bias in studies	18	Present assessments of risk of bias for each included study.	
Results of individual studies	19	For all outcomes, present, for each study: (a) summary statistics for each group (where appropriate) and (b) an effect estimate and its precision (e.g. confidence/credible interval), ideally using structured tables or plots.	
Results of syntheses	20a	For each synthesis, briefly summarise the characteristics and risk of bias among contributing studies.	
	20b	Present results of all statistical syntheses conducted. If meta-analysis was done, present for each the summary estimate and its precision (e.g. confidence/credible interval) and measures of statistical heterogeneity. If comparing groups, describe the direction of the effect.	
	20c	Present results of all investigations of possible causes of heterogeneity among study results.	
	20d	Present results of all sensitivity analyses conducted to assess the robustness of the synthesized results.	
Reporting biases	21	Present assessments of risk of bias due to missing results (arising from reporting biases) for each synthesis assessed.	
Certainty of evidence	22	Present assessments of certainty (or confidence) in the body of evidence for each outcome assessed.	
**DISCUSSION**			
Discussion	23a	Provide a general interpretation of the results in the context of other evidence.	
	23b	Discuss any limitations of the evidence included in the review.	
	23c	Discuss any limitations of the review processes used.	
	23d	Discuss implications of the results for practice, policy, and future research.	
**OTHER INFORMATION**			
Registration and protocol	24a	Provide registration information for the review, including register name and registration number, or state that the review was not registered.	
	24b	Indicate where the review protocol can be accessed, or state that a protocol was not prepared.	
	24c	Describe and explain any amendments to information provided at registration or in the protocol.	
Support	25	Describe sources of financial or non-financial support for the review, and the role of the funders or sponsors in the review.	
Competing interests	26	Declare any competing interests of review authors.	
Availability of data, code and other	27	Report which of the following are publicly available and where they can be found: template data collection forms; data extracted from included studies; data used for all analyses; analytic code; any other	

Section and Topic	Item #	Checklist item	Location where item is reported
materials		materials used in the review.	

Figure S1 Risk of bias assessment using Robvis[1]

	D1	D2	D3	D4	D5
Kim 2020	X	+	+	-	X
Leskela 2020	X	+	+	+	X
Fraune 2020	-	-	-	X	X
Carnevali 2019	+	-	-	-	-
Sugino 2019	+	+	+	-	+
Segev 2015	+	-	+	X	X
Geisler 2000	-	-	X	X	X
Stratton 1999	X	-	X	X	X
Schmoeckel 2019	X	-	+	-	X
Bennet 2019	X	-	-	X	X
Tajima 2018	+	+	+	X	-
Xue 2018	-	-	+	X	-
Akbari 2017	-	-	X	X	X
Bennet 2016	X	+	-	-	X
Zhai 2008	+	+	-	X	-
Shilpa 2014	+	+	-	X	-
Vierkoetter 2014	X	-	+	X	X
Song 2014	+	+	-	+	+
Lee 2014	+	+	-	X	-
Niskakowski 2013	+	-	X	X	X
Coppola 2012	+	+	-	-	+
Pal 2012	+	+	-	+	+
Lu 2012	-	+	+	-	-
Fuseya 2012	-	+	+	-	-
Permuth-Wey 2009	+	+	-	-	-
Helleman 2006	+	-	+	X	-
Malander 2006	-	-	+	-	-
Singer 2004	+	+	+	X	-
Arzimanoglou 1996	-	-	+	+	+
Koczkowska 2018	+	-	-	X	X
Leskela 2020b	X	-	+	-	X
Zhu 2019	X	+	+	-	X
Parra-Herran 2019	X	+	+	X	X
Yamashita 2019	-	+	-	+	+
Brandt 2017	+	-	X	-	-
Hirasawa 2017	+	-	+	X	-
Parra-Herran 2017	X	+	-	X	X
Wang 2017	+	-	+	-	+
Keleman 2017	X	-	+	X	X
Rambau 2016	+	-	+	X	-
Norquist 2016	+	-	X	-	-
Susswein 2016	+	-	-	-	-
Stewart 2013	X	+	+	+	X
South 2008	X	-	X	X	X
Domanska 2007	X	+	+	-	X
Geisler 2003	+	+	-	X	-
Rubin 1998	+	+	+	-	+
Harter 2017	+	-	+	X	-
Gras 2001	X	+	-	X	-
Catasus 2004	-	-	-	-	-
Liu 2004	X	-	-	X	-
Sood 2001	X	-	+	X	-
Gifford 2004	-	-	+	X	-

Judgement

X High

- Some concerns

+ Low

<u>Domains</u>

D1 = Selection

D2 = Comparability

D3 = Exposure

D4 = Outcome

D5 = Overall

Figure S2 Proportion of ovarian cancer with mismatch repair deficiency by immunohistochemistry as reported by individual studies

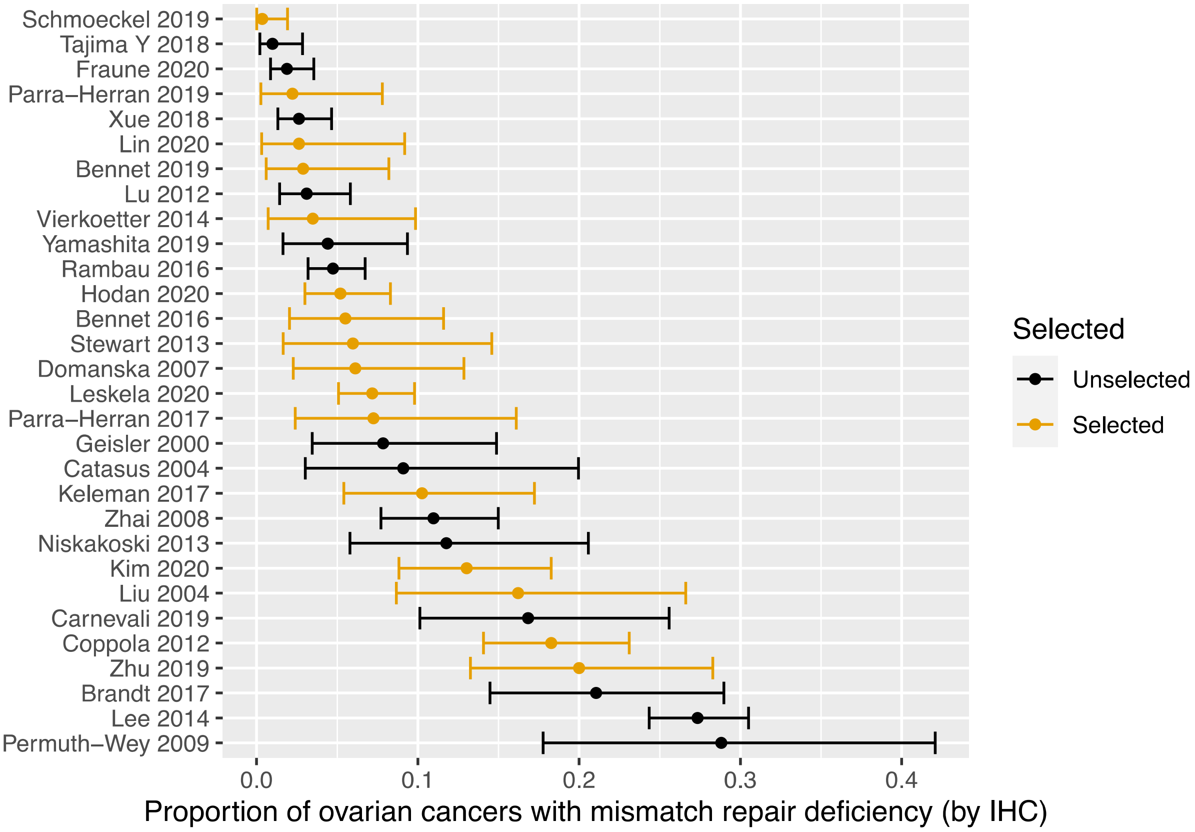

As shown in *Figure S2*, a total of 29 studies met the criteria for analysis, and these found a significant range of test positivity rates for immunohistochemistry, from 0.3% to 29%. There was no immediately obvious effect of selection of ovarian cancer cases (although it is possible that selection bias could act in different directions on the test positivity rate of IHC).

Figure S3 Proportion of ovarian cancer with mismatch repair deficiency by microsatellite instability analysis as reported by individual studies

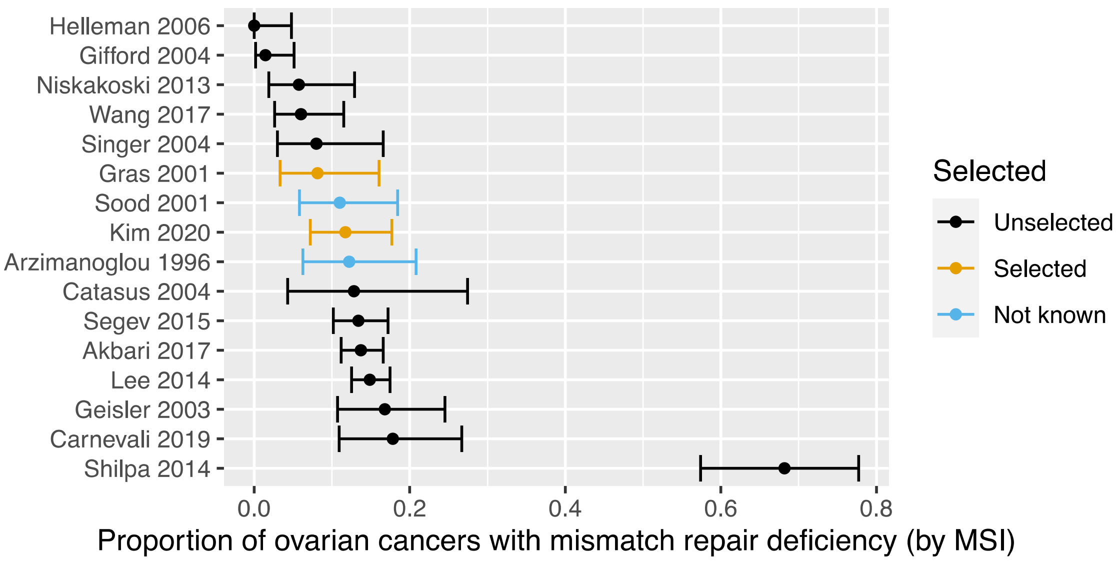

As shown in *Figure S3* a total of 15 studies met the criteria for analysis, and these found a significant range of test positivity rates for MSI, from 0% to 68%. Again, there was no immediately obvious effect of selection of ovarian cancer cases (although it is possible that selection bias could act in different directions on the test positivity rate of MSI). The study by Shilpa et al. 2014 appears to be a significant outlier. This study notably also found MSI in 47% of normal ovaries

Figure S4 Proportion of ovarian cancer with a germline path_MMR in Considering studies in which MSI and/or IHC were conducted

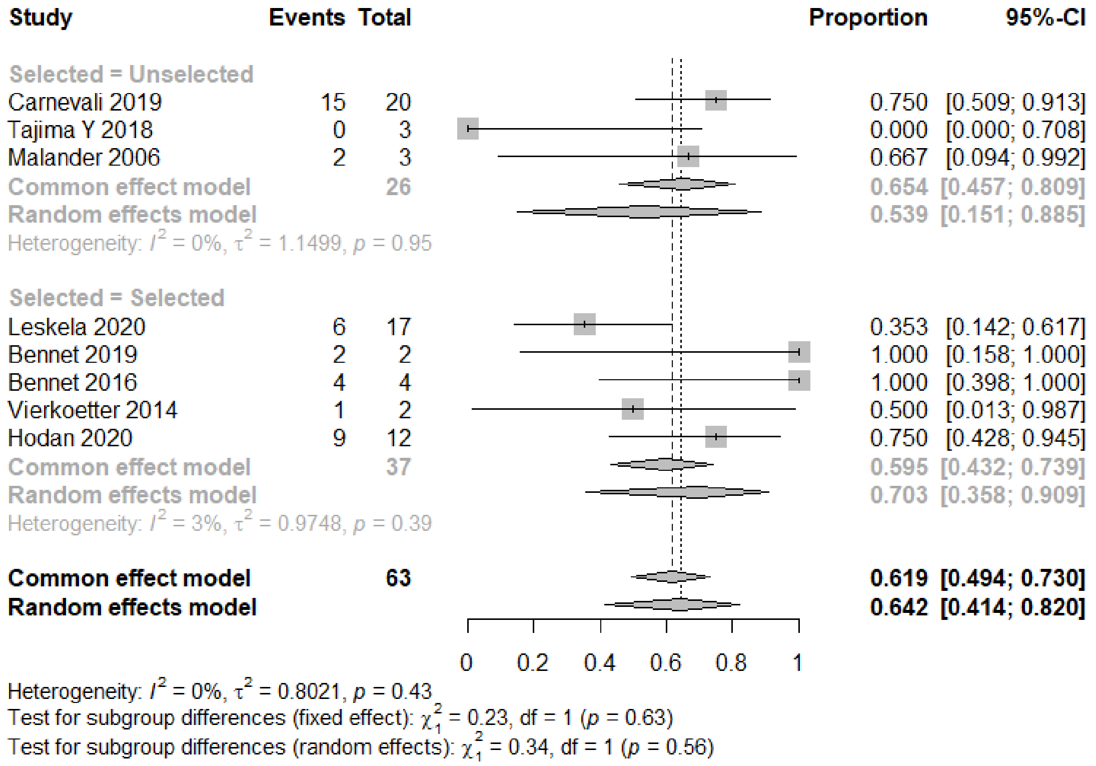

References:

1 McGuinness LA, Higgins JPT. Risk-of-bias VISualization (robvis): An R package and Shiny web app for visualizing risk-of-bias assessments. *Res Synth Methods* 2020;12:55–61. doi:10.1002/jrsm.1411
